# Acute effects of cold, heat and contrast pressure therapy on forearm muscles regeneration in combat sports athletes: a randomized clinical trial

**DOI:** 10.1038/s41598-024-72412-0

**Published:** 2024-09-28

**Authors:** Robert Trybulski, Adrian Kużdżał, Arkadiusz Stanula, Jarosław Muracki, Adam Kawczyński, Wacław Kuczmik, Hsing-Kuo Wang

**Affiliations:** 1 Provita Żory Medical Center, Żory, Poland; 2 Department of Medical Sciences, The Wojciech Korfanty Upper Silesian Academy, Katowice, Poland; 3 College of Medical Sciences, Institute of Health Sciences, University of Rzeszów, Rzeszów, Poland; 4 Laboratory of Sport Performance Analysis, Institute of Sport Sciences, Academy of Physical Education in Katowice, Katowice, Poland; 5 Institute of Physical Culture Sciences, Department of Physical Culture and Health, University of Szczecin, Szczecin, Poland; 6 Department of Paralympic Sport, Wroclaw University of Health and Sport Sciences, Wrocław, Poland; 7 Department and Clinic of General Surgery, Vascular Surgery, Angiology and Phlebology, Faculty of Medical Sciences in Katowice, Medical University of Silesia in Katowice, Katowice, Poland; 8 School and Graduate Institute of Physical Therapy, National Taiwan University, Taipei, Taiwan; 9 Center of Physical Therapy, National Taiwan University, Taipei, Taiwan

**Keywords:** Sports recovery, MMA, Game-ready therapy, Myotonometry, Microcirculation, Physiology, Musculoskeletal system, Muscle, Fatigue, Clinical trial design

## Abstract

Due to the specific loads that occur in combat sports athletes' forearm muscles, we decided to compare the immediate effect of monotherapy with the use of compressive heat (HT), cold (CT), and alternating therapy (HCT) in terms of eliminating muscle tension, improving muscle elasticity and tissue perfusion and forearm muscle strength. This is a single-blind, randomized, experimental clinical trial. Group allocation was performed using simple 1:1 sequence randomization using the website randomizer.org. The study involved 40 40 combat sports athletes divided into four groups and four therapeutic sessions lasting 20 min. (1) Heat compression therapy session (HT, n = 10) (2) (CT, n = 10), (3) alternating (HCT, n = 10), and sham, control (ShT, n = 10). All participants had measurements of tissue perfusion (PU, [non-reference units]), muscle tension (T—[Hz]), elasticity (E—[arb- relative arbitrary unit]), and maximum isometric force (Fmax [kgf]) of the dominant hand at rest (Rest) after the muscle fatigue protocol (PostFat.5 min), after therapy (PostTh.5 min) and 24 h after therapy (PostTh.24 h)**.** A two-way ANOVA with repeated measures: Group (ColdT, HeatT, ContrstT, ControlT) × Time (Rest, PostFat.5 min, PostTh.5 min, Post.24 h) was used to examine the changes in examined variables. Post-hoc tests with Bonferroni correction and ± 95% confidence intervals (CI) for absolute differences (△) were used to analyze the pairwise comparisons when a significant main effect or interaction was found. The ANOVA for PU, T, E, and Fmax revealed statistically significant interactions of Group by Time factors (*p* < 0.0001), as well as main effects for the Group factors (*p* < 0.0001; except for Fmax). In the PostTh.5 min. Period, significantly (*p* < 0.001) higher PU values were recorded in the HT (19.45 ± 0.91) and HCT (18.71 ± 0.67) groups compared to the ShT (9.79 ± 0.35) group (△ = 9.66 [8.75; 10.57 CI] > MDC_(0.73)_, and △ = 8.92 [8.01; 9.83 CI] > MDC_(0.73)_, respectively). Also, significantly (*p* < 0.001) lower values were recorded in the CT (3.69 ± 0.93) compared to the ShT (9.79 ± 0.35) group △ = 6.1 [5.19; 7.01 CI] > MDC_(0.73)_. For muscle tone in the PostTh.5 m period significantly (*p* < 0.001) higher values were observed in the CT (20.08 ± 0.19 Hz) group compared to the HT (18.61 ± 0.21 Hz), HCT (18.95 ± 0.41 Hz) and ShT (19.28 ± 0.33 Hz) groups (respectively: △ = 1.47 [1.11; 1.83 CI] > MDC_(0.845)_; △ = 1.13 [0.77; 1.49 CI] > MDC_(0.845)_, and △ = 0.8 [0.44; 1.16 CI], < MDC_(0.845)_). The highest elasticity value in the PostTh.5 m period were observed in the CT (1.14 ± 0.07) group, and it was significantly higher than the values observed in the HT (0.97 ± 0.03, △ = 0.18 [0.11; 0.24 CI] > MDC_(0.094)_, *p* < 0.001), HCT (0.90 ± 0.04, △ = 0.24 [0.17; 0.31 CI] > MDC_(0.094)_, *p* < 0.001) and ShT (1.05 ± 0.07, △ = 0.094 [0.03; 0.16 CI] = MDC_(0.094)_, *p* = 0.003) groups. For Fmax, there were no statistically significant differences between groups at any level of measurement. The results of the influence of the forearm of all three therapy forms on the muscles' biomechanical parameters confirmed their effectiveness. However, the effect size of alternating contrast therapy cannot be confirmed, especially in the PostTh24h period. Statistically significant changes were observed in favor of this therapy in PU and E measurements immediately after therapy (PostTh.5 min). Further research on contrast therapy is necessary.

## Introduction

Combat sports are characterized by intermittent high-intensity muscle work interspersed with short regeneration periods^1,2^. The fighter's fighting strategy and stylistic preferences determine the advantage of various types of strength and endurance during the fight^2^. Grappling and choking manoeuvres require the expression of isometric strength, considered a critical physical characteristic that distinguishes elite grapplers, while kicks and punches characterize attackers^3^. Therefore, the function of the forearm muscles plays a vital role in the fights of judo, jujitsu, and mixed martial arts (MMA) fighters^4,5^

Previous research has highlighted the need for high levels of dynamic and isometric strength (in both upper and lower extremities) to improve the performance of critical skills in combat sports^3^. This highlights the unique physical demands of combat sports and the crucial role of strength training in meeting these demands. Additionally, combat sports require appropriate training strategies, especially strength training strategies due to the physiological profile, and post-exercise regeneration strategies to counteract the adverse effects of physical exercise^5–7^.

Heavy physical loads may cause disturbances in the biomechanical parameters of muscles, post-exercise pain, and weakening of strength, which leads to functional limitations and may increase the risk of injury^8^. Muscle regeneration strategies after exercise can influence the recovery rate of lost muscle properties, and their role is mainly based on helping the human body release its natural self-repair potential through a series of complex physiological reactions^5,9^.

The use of various cold and heat therapies on sports recovery are a common practice among athletes and coaches of different sports, including combat sports^4,10,11^. However, the effectiveness of cold stimulus therapy^4,10,11^ and heat therapy^12,13^ produces conflicting results in terms of effectiveness. Conflicting results also suggest that cold stimuli may accelerate^14^ and impair^15^ regeneration similarly to heat stimuli^16,17^.

Recently, compression has been added to warm and cold stimuli, expecting a more effective regeneration-stimulating effect^16,18,19^. However, the results using heat and cold-enhanced compression are still in the research phase, and more research is needed to provide practical implications^20,21^. Studies have shown that cryo-compression reduces edema^22^ and improves tissue perfusion^7^, as does heat compression^16,23^. However, regarding perfusion, only the evidence regarding heat is conclusive^24^, while the evidence regarding the use of compression and cold is inconclusive^25^. Moreover, the literature indicates the analgesic effect of both stimuli and suggests a beneficial impact on post-exercise regeneration, muscle tension, and elasticity^22,26,27^.

Significantly, few studies have compared the effects of contrast therapy^28–30^. There are no RCTs; therefore, the most effective recovery protocol is unknown. We noticed a gap in the scientific literature in studies assessing the effectiveness of post-exercise muscle recovery therapy and comparing heat compression (HT) and cold compression (CT) with cold and heat contrast therapy (HCT).

Like cold stimuli, warm stimuli in sports medicine are common and frequent. The most crucial physiological parameter emphasized by the authors is blood flow, which is the primary carrier of heat removal from tissues^17^. This aspect causes the blood supply to the tissues to impact the ability to heat the tissues significantly. Numerous beneficial angiogenic effects of heat and improvement of microcirculatory function are observed^31^. The anti-inflammatory^32^ and the analgesic effect^33^ of heat are well described. In post-exercise muscle regeneration strategies, it is assumed that warm stimuli relax affect muscle tone and improve muscle elasticity and stiffness. However, there are still few research results in this area, most of which are secondary^34^. Kobayashi et al. suggest that a single session of whole-body heat stress in rodents increases muscle calcineurin expression regardless of fiber type^35^. Among the mechanisms by which heat stress induces an increase in muscle mass, apart from calcineurin, the scientific literature describes an increase in the activity of serine-threonine phosphatase. Interactions between relevant heat shock proteins after heat therapy and factors related to muscle hypertrophy are confirmed, but the mechanisms driving them remain unclear^36^. Among the factors regulating muscle mass, attention is paid to the effect of a thermal stimulus on the inhibition of protein degradation and mitochondrial clearance. Heat can delay muscle atrophy, but the effects are inconclusive^37^.

Alternating heat and cold stimuli are also popular in sports recovery^19^. Alternating water immersion at variable temperatures, used as partial immersion in hot (HWI) and cold water (CWI), is particularly well described^38^. This therapy consists of several cycles with a total time of 5 to 20 min^30^. The water temperature ranges from 3 to 15 °C for the cold stimulus and from 40 to 45 °C for the warm stimulus^39^. In addition to water immersion, there are other popular contrast therapy strategies, among which an innovative therapy gaining popularity is contrast compression therapy Game Ready (GR)^40^. GR therapy combines an alternating hot and cold stimulus that is applied to a specific tissue as a pressure cuff. The pressure we can apply is variable, ranging from 15 to 75 mmHg, and the temperature is from 3 to 45 (°C). The duration of this treatment varies from 10 to 30 min^41,42^.

Contrast therapy influences muscle tension and elasticity, leading to faster recovery of these parameters, especially after intense physical exercise^29^. Improving these parameters may be one of the factors reducing the risk of injury in combat sports^39^. In addition, biomechanical parameters such as stiffness, tension, and elasticity affect muscle power^43^, the ability to continue physical exercise, and muscle pain^30^.

The study aimed to compare the effectiveness of HT and CT monotherapy and HCT contrast therapy (cold and heat) using the Game Ready device regarding muscle tension, elasticity, tissue perfusion, and muscle strength. The research hypothesis assumed that intervention with HCT may be a better option for muscle recovery without injuries than intervention with heat or cold alone. We hypothesized that HCT therapy would show more favorable changes in the measured parameters and help optimize regeneration processes in sports. Assessment of musculoskeletal tissue and its properties before and after applying various stimuli is crucial in preventing overuse injuries associated with repeated exposure to low or high levels of force. The study's results may provide general information necessary to develop practical recovery strategies in combat sports. Intentional significance of the study for the researchers is to bring evidence on the impact of heat and cold monotherapy and combined heat-cold therapy; for clinicians—to deliver evidence on how to construct the recovery protocols; and for the patients—the provide results which justify using the best method of therapy in sports recovery.

## Methods

### Study design

**Fig. 1:**
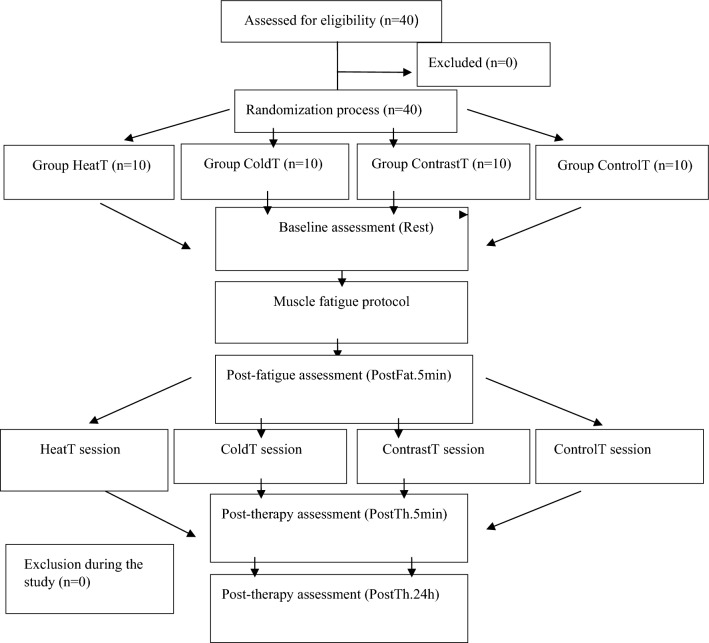
Study design.

Forty combat training volunteers (n = 40) participated in this prospective, single-blind, randomized clinical trial. The participants were recruited in May 2023 and randomly divided into four groups (A, B, C, D) with different therapeutic interventions (sessions): A—heat compression therapy (HeatT, n = 10), B—cold compression therapy (ColdT, n = 10), C—cold and heat contrast therapy (ContrastT, n = 10) and D—sham therapy as control (controlT, n = 10) (Fig. 1). The sequence of experimental procedures:1 Resting measurement, 2 muscle fatigue protocols, 3 measurements after muscle fatigue, 4 regeneration interventions, 5 immediate measurements after regeneration intervention, 6 measurements after 24-h regeneration. The measurements and intervention were taken between 5 and 7 June 2023. All participants were tested between 9 am and 2 pm at the Provita Medical Center in Poland. Before starting the research, volunteers completed a consent form and a health questionnaire. The same conditions prevailed during the tests: air temperature 21 °C and air humidity 45–50%). The experimental conditions were controlled by thermo-hygrometers, which are part of the equipment of the Provita Medical Center, where the research was carried out.

One of the authors (RT) performed a group assignment using a simple 1:1 randomization with a random sequence using the randomizer.org website. Group assignment was independent of treatment duration and study personnel. In addition, through randomization, the participant was assigned a session during which a specific or sham therapy was performed (participants were not informed about the type of therapy used, and due to lack of experience in this therapy, they could not assess which one was real and which one was sham). Data were collected by two experienced researchers who were blinded to which intervention participants were randomly assigned to. Each participant underwent a familiarization intervention, receiving 10 min of Game Ready stimulation seven days before the study. The study was approved by the ethics committee of the National Council of Physiotherapists (No. 9/22 of 6 April 2022) and registered in the clinical trials register on 25/05/2023 under the number https://doi.org/10.1186/ISRCTN90040217 and conducted by the Declaration of Helsinki.

### Muscle fatigue protocol

After calculating the maximum isometric strength of the forearm muscles (average of 3 measurements) (Fmax), the study participants used a modified test specific to the forearm muscles described in the literature^44,45^. The test required participants to maintain an isometric handgrip strength of 60% (± 10%) of their maximum voluntary contraction force on an electronic dynamometer during a 5-s exercise and a 2-s rest cycle. Each participant performed five repetitions before refusing to exercise, with a one-minute break. The breaks and the number of repetitions were supposed to resemble the rules of MMA fighting. The study participants were sitting with their upper limbs bent at the elbow joint at an angle of 90 degrees^46^. To prevent fatigue of the synergistic muscles of the shoulder girdle, the limb was supported on a couch^7^. The participants controlled the strength of their handgrip under the supervision of an assistant (an experienced physiotherapist) taking part in the survey. Additionally, medical staff, including a qualified paramedic, were available at the Provita Medical Center during the study.

### Regeneration intervention

**Fig. 2:**
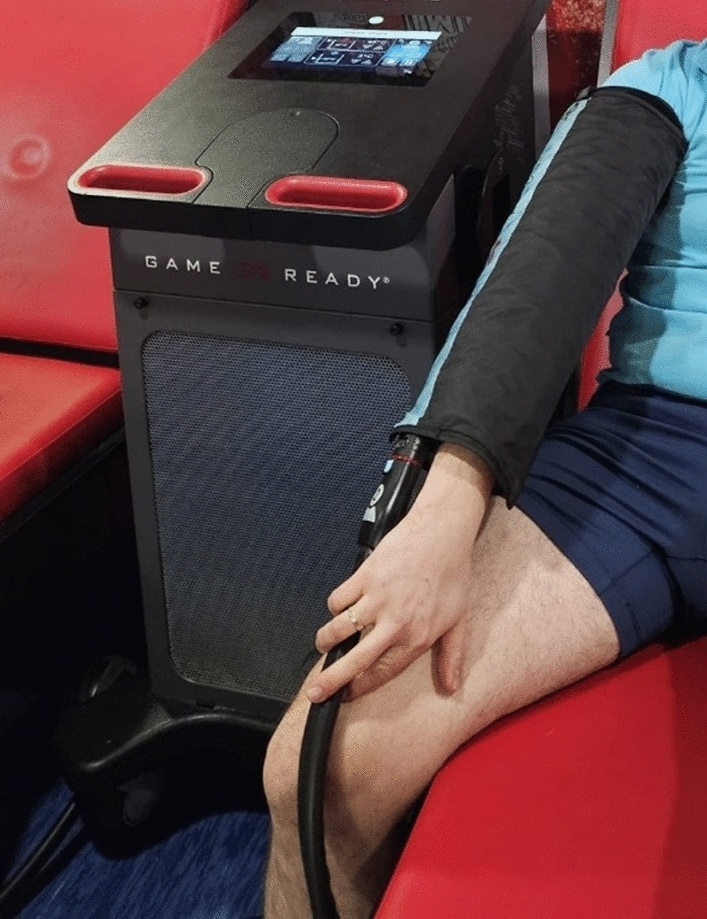
Game Ready contrast therapy device.

The device performed the following Game Ready applications alternately through a cuff placed on the dominant forearm (Fig. 2):

1. HeatT (HT) session in which GR 1-min heat stimulation was applied for 20 min with a 1-min break, during which the cuff applied increasing pressure from 15 to 25 mmHg and a constant temperature of 45 °C.
2. ColdT (CT) session in which GR 1-min cold stimulation was applied for 20 min with a 1-min break, during which the cuff applied increasing pressure from 25 to 75 mmHg and a constant temperature of 3 °C.
3. ContrastT (HCT) (session, in which 20 min of alternating GR, 1 min of cold stimulation, and 1 min of heat therapy were applied, the cuff applied an increasing pressure from 25 to 75 mmHg and a temperature of 3 °C and 45 °C.
4. ControlT (ShT) control session during which 20 min sham therapy, 1 min GR stimulation was used followed by a 1-min break during which the cuff was applied, using the pressure of 15 mmHg and the temperature of 35 °C. The intervention protocol was developed based on contemporary literature^40,41^. After the intervention, measurements were collected in the same manner as at rest.

### Participants

Forty combat sports fighters (n = 40) volunteers were selected according to the following inclusion criteria: age 18–40 years old, gender male only, minimum of five years of combat sport training experience, and training at least four times per week. Considering McKay's participant classification scheme, the group of competitors belonged to Tier 2 and 3: Highly Trained/National Level^47^. Of the 40 volunteers, 22 declared mixed martial arts (MMA, n = 22) as their primary sport, ten judo (J, n = 10), and 8 Brazilian jiu-jitsu (BJJ, n = 8) (Table 1). Exclusion criteria were elevated blood pressure before the study (> 140/90 mmHg), currently treated injuries, damaged skin, or unspecified skin lesions at the measurement sites. Participants with tattoos at the measurement site were also excluded as it would interfere with tissue perfusion measurements. Exclusions were also made in the case of extreme fatigue, fever, infection, or at the explicit request of the participant. Written informed consent was obtained from participants after they were made aware of the study aims, risks and conditions. Participants were required to refrain from training 48 h before and for 48 h during the study. In addition, due to tissue perfusion measurements, participants were asked to refrain from consuming ergogenic beverages for 4 h before the study (list of forbidden drinks were delivered to every participant before the study). Participants could be excluded from the study at their request at any time during the study^7^.

**Table 1:** Random distribution of respondents representing disciplines in individual groups: cold therapy, heat therapy, contrast therapy, control therapy.

Group	ColdT	HeatT	ContrastT	ControlT	Total
MMA	5	5	6	6	22
JUDO	3	3	2	2	10
BJJ	2	2	2	2	8
Number	10	10	10	10	40

*MMA* mixed martial art; *BJJ* Brazilian jujitsu.

### Assessments

**Fig. 3:**
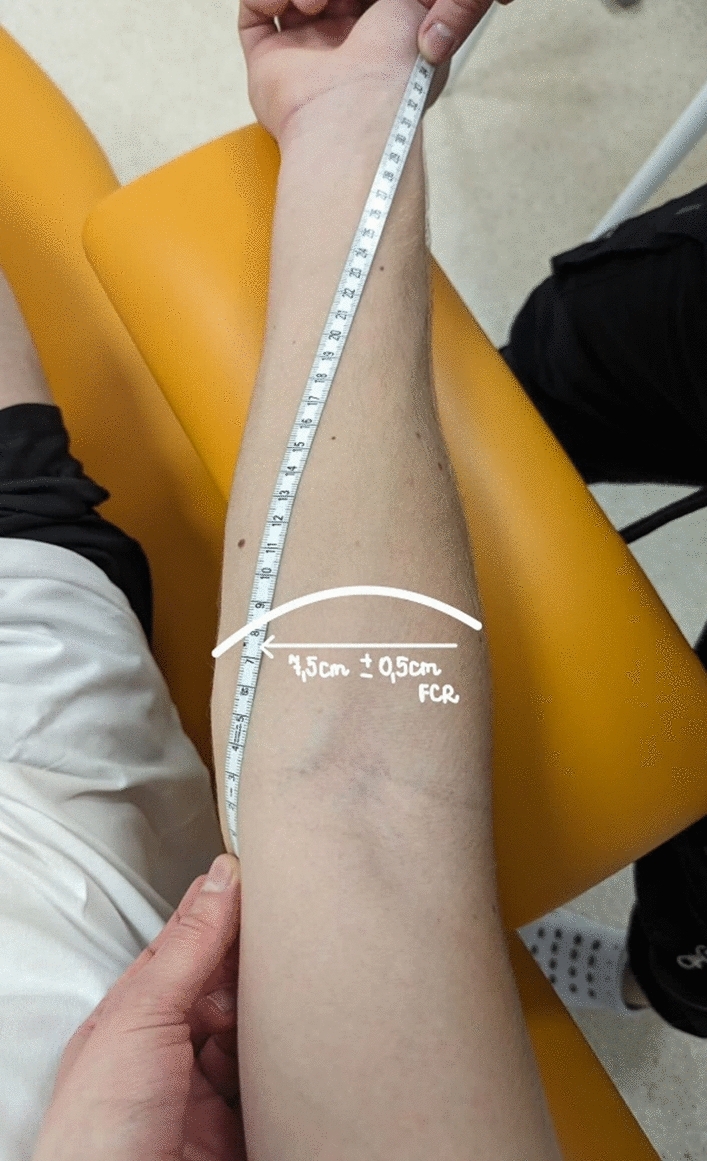
Marking the measurement point for flexor carpi radialis muscle (FCR).

The Accuniq BC720 body composition analyzer (South Korea 2019) was used to assess the anthropometric characteristics of the study participants. The random division into groups was conducted, and afterward, the groups were checked and confirmed to not differ in terms of the anthropometric characteristics of research participants (Table 2). After determining and marking the broadest cross-sectional area of the flexor carpi radialis muscle (FCR) of the dominant hand under ultrasound guidance with a marker, the following assessment was taken in all participants^7^ (Fig. 3): microvascular response described in non-reference units (PU), muscle tone (T—[Hz]), elasticity (E—[arb- relative arbitrary unit]), and maximum isometric force (Fmax [kgf]). The study aimed to assess immediate effects, so the scientific literature and our previous research adopted the measurement periods mentioned^5,16^. All participants were tested in a standardized resting position described in the previous paragraphs (a sitting position with their upper limbs bent at the elbow joint at an angle of 90 degrees). All assessments were performed during the indicated periods: rest, 1–5 min after the fatigue protocol (PostFat.5 min.), 5 min after therapy (PostTh5min.), and 24 h after treatment (PostTh24h). The choice of these measurement periods is related to the study's purpose, i.e., immediate assessment of changes, and is not arbitrary. It is a deliberate alignment with previous studies of the acute effects of regeneration, providing a solid foundation for our research methodology^6,7,16^. In this article which is a part of a bigger project one recovery session was analyzed^16^. Appropriately trained physiotherapists took part in the assessment. The measurements were taken in the following order: (1) perfusion unit, (2) muscle tone, (3) elasticity, (4) and Fmax.

**Table 2:** Characteristics of research participants in terms of anthropometric data and training experience groups: cold therapy, heat therapy, contrast therapy, control.

	ColdT	HeatT	ContrastT	ControlT	F	p
Age (years)	25.80 ± 3.33	29.10 ± 5.13	27.10 ± 3.84	28.20 ± 4.21	1.162	0.338
Height (cm)	177.30 ± 9.09	182.80 ± 5.35	178.70 ± 4.88	181.20 ± 7.48	1.274	0.298
Weight (kg)	78.06 ± 9.22	83.41 ± 11.11	84.67 ± 9.36	82.39 ± 9.68	0.846	0.478
Training experience (years)	10.20 ± 5.35	11.80 ± 4.16	10.70 ± 4.22	10.80 ± 4.26	0.219	0.882
BMI(kg m^-1^)	24.47 ± 1.87	25.84 ± 3.92	26.00 ± 3.09	26.07 ± 2.85	0.626	0.603

There were no statistically significant differences between the groups for all analyzed variables (*p* > 0.05).

#### Perfusion unit (PU)

Laser Doppler flowmetry (LDF) was used for PU analysis (Permitted, Sweden 2004). The wave reflected from the erythrocytes was measured after clinically determining the place and marking it with a special pen^48^. The measurement depth was 2.5 mm, the volume was one mm3, and the measurement in a standardized position lasted 2 min^49^. The LDF method, thanks to its repeatability and validity, high sensitivity, and non-invasive nature, allows for a precise assessment of microcirculation at rest and in response to a physical stimulus^48^. LDF is a standard and reproducible technology enabling the study of microcirculatory function in vivo^50^. Better sensors and more effective wireless communication interest scientists and clinicians.

#### Myotonometry

To assess the biomechanical parameters of the FCR muscle (T, S, and E), a myotonometer was used—MyotonPRO (Myoton, Estonia), which is a digital palpation device consisting of a body and a depth probe (Ø 3 mm)^51^. MyotonPro uses the Mechanical Dynamic Response method, which involves a precise mechanical impulse, recording the dynamic response of the tissue in the form of a signal of physical displacement and oscillation acceleration, and then calculating parameters characterizing the tested biomechanical properties^52^. Its reliability and repeatability have been confirmed in scientific literature^53^. The probe is applied perpendicular to the tissue, with an initial pressure of approximately (0.18 N) and the device releases a mechanical pulse (0.4 N, 15 ms), deforming the tissue for a short time^52^. The algorithm calculates tissue oscillations, the amount of its deformation, the time to return to the rest position, and the damping of tissue vibrations, determining relative values for specific parameters^52^. The total measurement time was up to 15 s.

#### Muscle force

Maximum forearm muscle force (Fmax) was the last measurement taken at rest and after stimulation. An electronic hand dynamometer measured the maximum forearm muscle force (Fmax) (EH106, China). The measurement was performed in. The maximum forearm muscle force expressed in kg was calculated based on this. The test–grip strength consisted of a 5-s contraction of the forearm muscles while squeezing the dynamometer^7^. Before the test, each participant performed a warm-up that involved exerting maximum pressure on a small ball ten times, followed by a 10-s stretching of the forearm muscles. Stretching is a standard warm-up procedure^5^. To prevent muscle damage during contraction. A one-minute break after stretching was taken before the tests to avoid the influence of pre-stretching on contraction strength. This instrument (dynamometer) is widely used to assess isometric strength and demonstrates high reliability and validity in repeatable measurements^54,55^. The total measurement time was up to 15 s.

### Statistical analysis

Means and standard deviations were used to represent the average and the typical spread of all measured variables. The normal distribution of the data was verified using the Shapiro–Wilk test. Homoscedasticity was tested using the Levene test. Compound symmetry, or sphericity, was checked using the Mauchly test. When the assumption of sphericity was not met, the significance of F-ratios was adjusted according to the Greenhouse–Geisser procedure. A two-way ANOVA with repeated measures: Group (ColdT, HeatT, ContrstT, ControlT) × Time (Rest, PostFat.5 min, PostTh.5 min, Post.24 h) was used to examine the changes in examined variables (Table 3). Post-hoc tests with Bonferroni correction and ± 95% confidence intervals (CI) for absolute differences (△) were used to analyze the pairwise comparisons when a significant main effect or interaction was found. Effect sizes were calculated using partial eta squared (η_p_^2^) and interpreted according to the following criteria: if 0 ≤ η_p_^2^ < 0.05, there is no effect; if 0.05 ≤ η_p_^2^ < 0.26, the effect is minimal; if 0.26 ≤ η_p_^2^ < 0.64, the effect is moderate; and if η_p_^2^ ≥ 0.64, the effect is strong^56^. Furthermore the Minimal Detectable Change (MDC) was used to determine whether the observed effects are clinically significant. A priori power analysis was conducted with the program G ∗ Power^57^. The repeated measure ANOVA within-between interactions with an effect size of at least 0.25, α = 0.05, and 1-β = 0.95 gave a statistical power of 97.2% and the total sample size of 40 subjects. All statistical analyses were performed using Statistica 13.3 (TIBCO Software Inc., Palo Alto, CA, USA, 2017). Statistical significance was set at *p* < 0.05.

**Table 3:** Average values for the studied groups in subsequent measurement periods. Data are expressed as means ± standard deviations.

Variables	Group	Rest	PostFat.5 min	PostTh.5 min	PostTh.24 h
PU	ColdT	8.20 ± 0.94	15.02 ± 1.06	3.69 ± 0.93	7.92 ± 0.76
	HeatT	8.48 ± 0.78	14.64 ± 0.93	19.45 ± 0.91	10.01 ± 0.41
	ContrastT	8.65 ± 0.60	13.54 ± 0.84	18.71 ± 0.67	10.93 ± 0.43
	ControlT	8.63 ± 0.65	14.34 ± 0.93	9.79 ± 0.35	9.69 ± 0.22
Fmax	ColdT	52.13 ± 6.64	42.92 ± 4.62	46.43 ± 5.31	47.97 ± 4.75
	HeatT	51.79 ± 5.92	42.92 ± 5.39	45.01 ± 4.63	46.58 ± 4.36
	ContrastT	52.13 ± 5.38	43.60 ± 4.57	45.72 ± 4.05	46.54 ± 4.19
	ControlT	52.16 ± 5.82	43.52 ± 4.47	43.84 ± 3.86	50.16 ± 4.93
T	ColdT	18.95 ± 0.44	19.63 ± 0.35	20.08 ± 0.19	17.80 ± 0.48
	HeatT	18.32 ± 0.59	19.53 ± 0.43	18.61 ± 0.21	17.39 ± 0.36
	ContrastT	18.83 ± 0.55	19.62 ± 0.35	18.95 ± 0.41	18.45 ± 0.68
	ControlT	17.98 ± 0.43	19.36 ± 0.33	19.28 ± 0.33	18.81 ± 0.28
E	ColdT	0.89 ± 0.03	0.99 ± 0.07	1.14 ± 0.07	0.89 ± 0.04
	HeatT	0.94 ± 0.03	1.11 ± 0.07	0.97 ± 0.03	0.88 ± 0.03
	ContrastT	0.89 ± 0.03	1.13 ± 0.08	0.90 ± 0.04	0.89 ± 0.03
	ControlT	0.93 ± 0.02	1.10 ± 0.05	1.05 ± 0.07	0.98 ± 0.01

Perfusion unit (PU, [non-reference units]), muscle tone (T—[Hz]), elasticity (E—[arb- relative arbitrary unit]), and maximum isometric force (Fmax [kgf]).

## Results

**Fig. 4:**
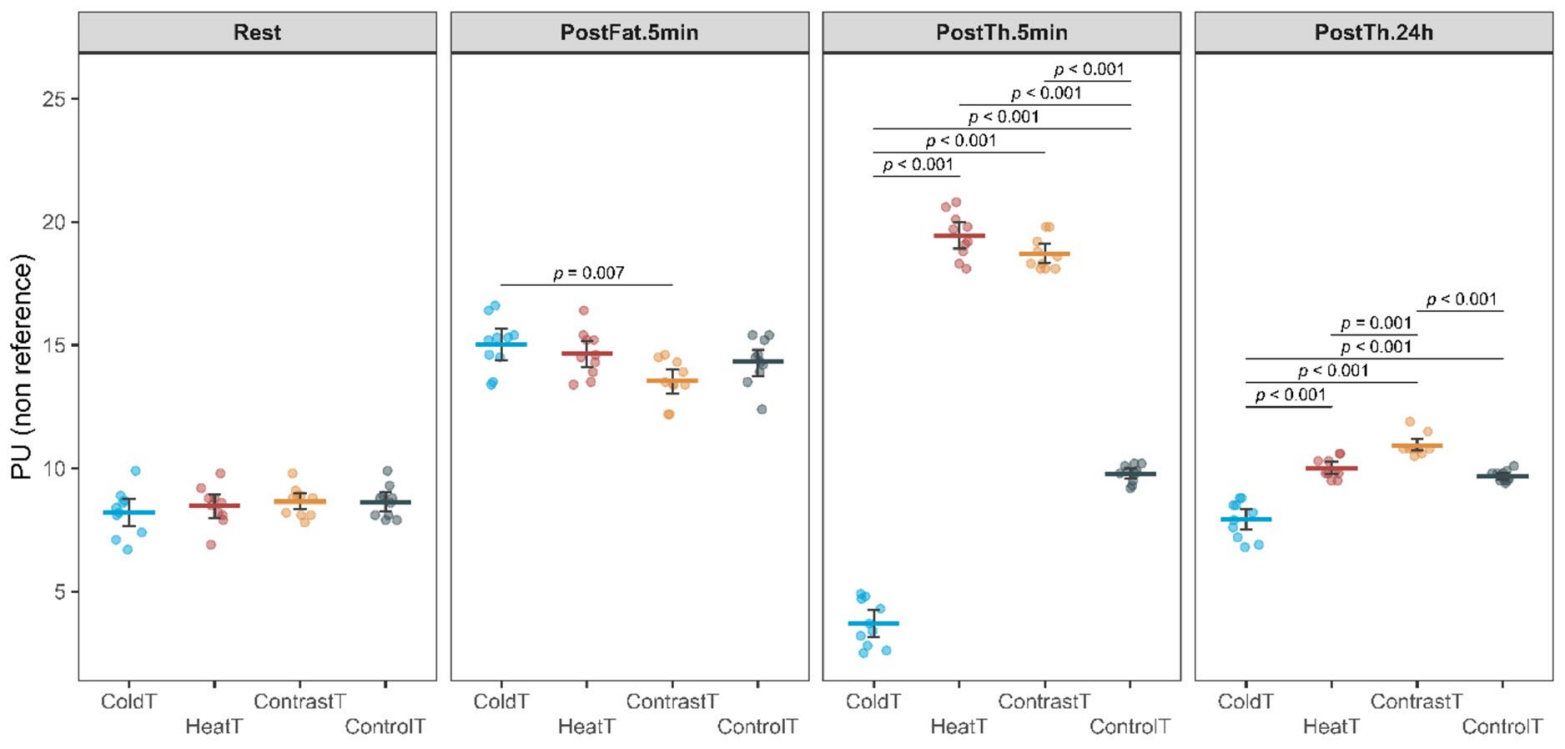
Change in Perfusion Unit for all measurement sessions in HeatT, ColdT, ContrastT and ControlT groups.

Analysis of variance for PU revealed a significant interaction of Group by Time factors (F_(7.3, 87.4)_ = 257.25, *p* < 0.001, η_p_^2^ = 0.94), as well as main effects for Group factors (F_(3, 36)_ = 264.22, *p* < 0.001, η_p_^2^ = 1.00) and Time (F_(2.4, 87.4)_ = 566.99, *p* < 0.001, η_p_^2^ = 0.96). In the PostFat.5 min. period, a significant difference was observed between the ColdT and ContrastT groups (15.02 ± 1.06 vs. 13.54 ± 0.84, △ = 1.48 [0.34; 2.62 CI] > MDC_(0.73)_, *p* = 0.007) (Fig. 4). In the PostTh.5 min. period, significantly higher PU values were recorded in the HeatT, ContrastT, and ControlT groups compared to the ColdT group (respectively: 19.45 ± 0.91 vs. 3.69 ± 0.93, △ = 15.76 [14.85; 16.67 CI] > MDC_(0.73)_, *p* < 0.001; 18.71 ± 0.67 vs. 3.69 ± 0.93, △ = 15.02 [14.11; 15.93 CI] > MDC_(0.73)_, *p* < 0.001, and 9.79 ± 0.35 vs. 3.69 ± 0.93, △ = 6.1 [5.19; 7.01 CI] > MDC_(0.73)_, *p* < 0.001). Also, significantly higher values were recorded in the HeatT and ContrastT groups compared to the ControlT group (respectively: 19.45 ± 0.91 vs. 9.79 ± 0.35, △ = 9.66 [8.75; 10.57 CI] > MDC_(0.73)_, *p* < 0.001 and 18.71 ± 0.67 vs. 9.79 ± 0.35, △ = 8.92 [8.01; 9.83 CI] > MDC_(0.73)_, *p* < 0.001). In turn, after 24 h (PostTh.24), significantly higher values were observed in the HeatT, ContrastT, and ControlT groups compared to the ColdT group (respectively: 10.01 ± 0.41 vs. 7.92 ± 0.76, △ = 2.09 [1.49; 2.69 CI] > MDC_(0.73)_, *p* < 0.001; 10.93 ± 0.43 vs. 7.92 ± 0.76, △ = 3.01 [2.41; 3.61 CI] > MDC_(0.73)_, *p* < 0.001, and 9.69 ± 0.22 vs. 7.92 ± 0.76, △ = 1.77 [1.17; 2.37 CI] > MDC_(0.73)_, *p* < 0.001). During this period, the highest PU value was recorded in the ContrastT group, which was significantly higher than in the HeatT and ControlT (respectively: 10.93 ± 0.43 vs. 10.01 ± 0.41, △ = 0.92 [0.32; 1,52 CI] > MDC_(0.73)_, *p* = 0.001, and 10.93 ± 0.43 vs. 9.69 ± 0.22, △ = 1.24 [0,64; 1,84 CI] > MDC_(0.73)_, *p* < 0.001) groups.

**Fig. 5:**
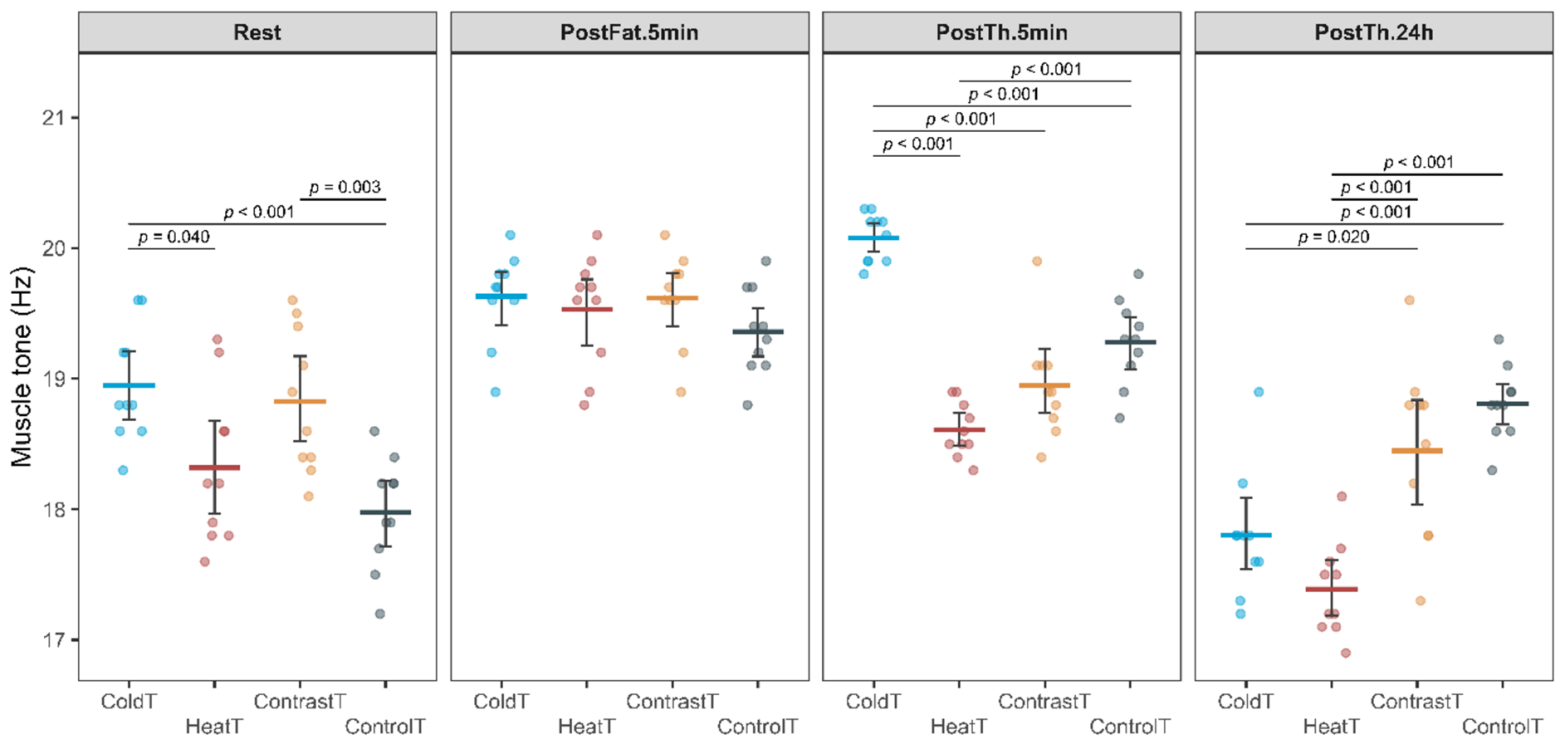
Change in Muscle Tone for all measurement sessions in HeatT, ColdT, ContrastT and ControlT groups.

Analysis of variance for muscle tone detecting an interaction of the Group by Time factors (F_(7.6, 90.7)_ = 21.61, *p* < 0.001, η_p_^2^ = 0.81), as well as main effects for the Group factors (F_(3, 36)_ = 8.42, *p* < 0.001, η_p_^2^ = 1.00) and Time (F_(2.5, 90.7)_ = 153.83, *p* < 0.001, η_p_^2^ = 0.41). In the Rest period, significantly higher values were observed in the ColdT group compared to the HeatT and ControlT groups (18.95 ± 0.44 Hz vs. 18.32 ± 0.59 Hz, △ = 0.63 [0.02; 1.24 CI] < MDC_(0.85)_, *p* = 0.040 and 18.95 ± 0.44 Hz vs. 17.98 ± 0.43 Hz, △ = 0.97 [0.36; 1.58 CI] > MDC_(0.845)_, *p* < 0.001, respectively), as well as higher values were observed in the ContrastT group compared to the ControlT group (18.83 ± 0.55 Hz vs. 17.98 ± 0.43 Hz, △ = 0.85 [0.24; 1.46 CI] > MDC_(0.845)_, *p* = 0.003. In the PostTh.5 min. period occurrence of differences between the HeatT and ContrastT groups (14.64 ± 0.93 vs. 13.54 ± 0.84, △ = -0.34 [-0,7; 0,02]*p* = 0.007) (Fig. 5). In the PostTh.5 m period, significantly higher muscle tone values were observed in the ColdT group compared to the HeatT, ContrastT and ControlT groups (20.08 ± 0.19 Hz vs. 18.61 ± 0.21 Hz, △ = 1.47 [1.11; 1.83 CI] > MDC_(0.845)_, *p* < 0.001; 20.08 ± 0.19 Hz vs. 18,0.95 ± 0.41 Hz, △ = 1.13 [0.77; 1.49 CI] > MDC_(0.845)_, *p* < 0.001, and 20.08 ± 0.19 Hz vs. 19.28 ± 0.33 Hz, △ = 0.8 [0.44; 1.16 CI], < MDC_(0.845)_, *p* < 0.001). The lowest muscle tone value in this period was recorded in the HeatT group and was significantly lower compared to the ControlT group (18.61 ± 0.21 Hz vs. 19.28 ± 0.33 Hz, △ = -0.67 [-1.03; -0.31 CI] < MDC_(0.845)_, *p* < 0.001). After 24 h (PostTh.24), the lowest values were observed in the HeatT group, which were significantly different from those observed in the ContrastT and ControlT groups (17.39 ± 0.36 Hz vs. 18.45 ± 0.68 Hz, △ = -1.06 [-1.63; -0.49 CI] > MDC_(0.845)_, *p* < 0.010, and 17.39 ± 0.36 Hz vs. 18.81 ± 0.28 Hz, △ = -1.42 [-1.99; -0.85] > MDC_(0.845)_, *p* < 0.001, respectively). Slightly higher values than the HeatT group were observed in the ColdT group, which were significantly lower than those observed in the ContrstT and ControlT groups (17.80 ± 0.48 Hz vs. 18.45 ± 0.68 Hz, △ = -0.65 [-1.22; -0.08 CI] < MDC_(0.845)_, *p* = 0.020, and 17.80 ± 0.48 Hz vs. 18.81 ± 0.28 Hz, △ = -1.01 [-1.58; -0.44] > MDC_(0.845)_, *p* < 0.001, respectively).

**Fig. 6:**
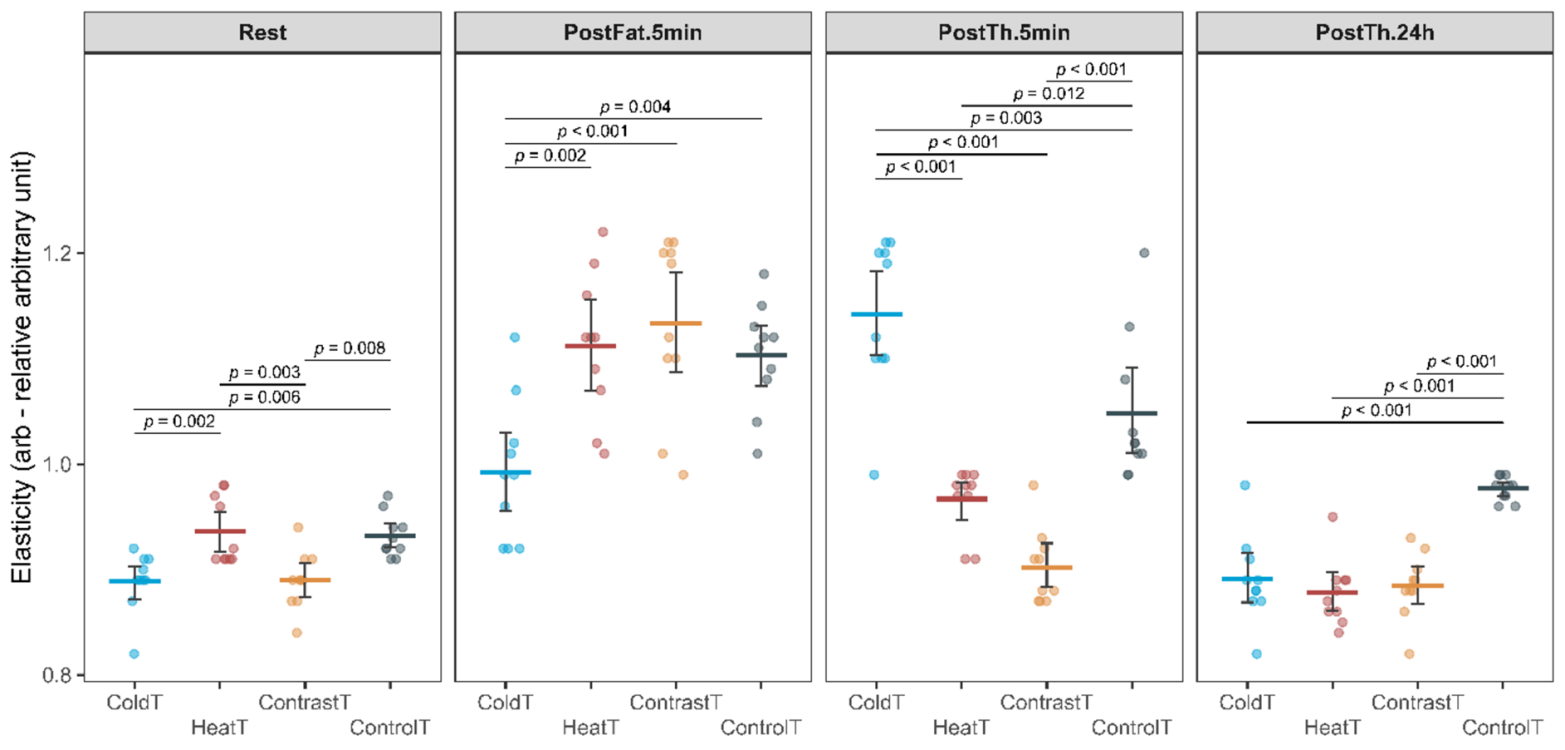
Change in elasticity for all measurement sessions in HeatT, ColdT, ContrastT and ControlT groups.

In the case of elasticity, the ANOVA revealed a significant interaction of the Group by Time factors (F_(6.7, 80.5)_ = 22.18, *p* < 0.001, η_p_^2^ = 0.79), as well as main effects for the Group factors (F_(3, 36)_ = 10.32, *p* < 0.001, η_p_^2^ = 1.00) and Time (F_(2.2, 80.5)_ = 131.78, *p* < 0.001, η_p_^2^ = 0.46). During the Rest period, significantly higher values were observed in the HeatT group compared to the ColdT and ContrastT groups (0.94 ± 0.03 vs. 0.89 ± 0.03, △ = 0.05 [0.01; 0.08 CI] < MDC_(0.094)_, *p* = 0.002, and 0.94 ± 0.03 vs. 0.89 ± 0.03△ = 0.05 [0.01; 0.08 CI] < MDC_(0.094)_, *p* = 0.003, respectively) (Fig. 6). Also, in the ControlT group, higher values were observed compared to the ColdT and ContrastT groups (0.93 ± 0.02 vs. 0.89 ± 0.03, △ = 0.04 [0.01; 0.08 CI] < MDC_(0.094)_, *p* = 0.006, and 0.93 ± 0.02 vs. 0.89 ± 0.03, △ = 0.04 [0.01; 0.07 CI] < MDC_(0.094)_, *p* = 0.008, respectively). In the PostFat.5 m period, the lowest elasticity value was recorded in the ColdT group, and it was significantly lower than the HeatT (0.99 ± 0.07 vs. 1.11 ± 0.07, △ = -0.12 [-0.2; -0.04 CI] > MDC_(0.094)_, *p* = 0.002), ContrastT (0.99 ± 0.07 vs. 1.13 ± 0.08, △ = -0.14 [-0.22; -0.06 CI] > MDC_(0.094)_, *p* < 0.001), and ControlT (0.99 ± 0.07 vs. 1.10 ± 0.05, △ = -0.11 [-0.19; -0.03 CI] > MDC_(0.094)_, *p* = 0.004) groups. Five minutes after the end of therapy (PostTh.5 min.), the highest elasticity value was observed in the ColdT group, and it was significantly higher than the values observed in the HeatT (1.14 ± 0.07 vs. 0.97 ± 0.03, △ = 0.18 [0.11; 0.24 CI] > MDC_(0.094)_, *p* < 0.001), ContrastT (1.14 ± 0.07 vs. 0.90 ± 0.04, △ = 0.24 [0.17; 0.31 CI] > MDC_(0.094)_, *p* < 0.001) and ControlT (1.14 ± 0.07 vs. 1.05 ± 0.07, △ = 0.094 [0.03; 0.16 CI] = MDC_(0.094)_, *p* = 0.003) groups. It should also be noted that the elasticity values in the HeatT and ContrastT groups were significantly lower than those in the ControlT group (0.97 ± 0.03 vs. 1.05 ± 0.07, △ = -0.08 [-0.15; -0.01] < MDC_(0.094)_, *p* = 0.012 and 0.90 ± 0.04 vs. 1.05 ± 0.07, △ = -0.15 [-0.21; -0.08] > MDC_(0.094)_, *p* < 0.001, respectively). After 24 h (PostTh.24), significantly higher values were observed in the ControlT group than in ColdT (0.98 ± 0.01 vs. 0.89 ± 0.04, △ = 0.09 [0.05; 0.12 CI] < MDC_(0.094)_, *p* < 0.001), HeatT (0.98 ± 0.01 vs. 0.88 ± 0.03, △ = 0.10 [0.06; 0.14 CI] > MDC_(0.094)_, *p* < 0.001) and ContrastT (0.98 ± 0.01 vs. 0.89 ± 0.03, △ = 0.09 [0.06; 0.13 CI] < MDC_(0.094)_, *p* < 0.001) groups.

**Fig. 7:**
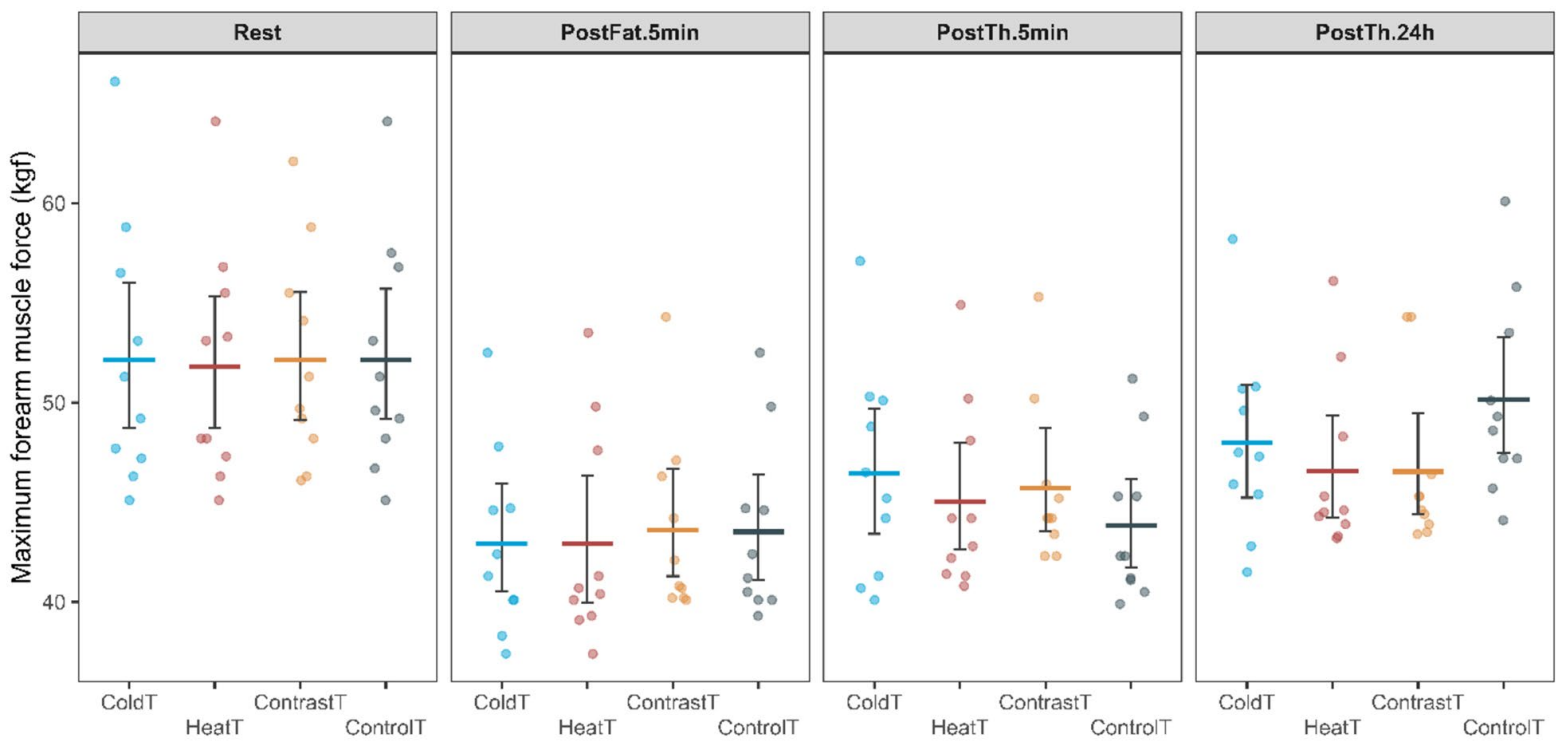
Change in maximum forearm muscle force for all measurement sessions in HeatT, ColdT, ContrastT and ControlT groups.

For maximum forearm muscle force, the ANOVA revealed a significant interaction of the Group by Time factors (F_(5.5, 65.5)_ = 3.66, *p* = 0.004, η_p_^2^ = 0.83) and the main effect of the Time factor (F_(1.8, 65.5)_ = 173.05, *p* < 0.001, η_p_^2^ = 0.01), with no significance for the main effect of the Group factor (F_(3, 36)_ = 0.07, *p* = 0.976, η_p_^2^ = 0.99) (Fig. 7).

## Discussion

Our research aimed to assess the impact of various forms of compression therapy using hot and cold stimuli and alternating therapy called contrast therapy on muscle tension, muscle elasticity, tissue perfusion, and maximum isometric strength. The study's main findings confirm statistically significant assessment parameter changes compared to the control group. However, the results did not confirm our hypothesis about a more effective contrast therapy. Only the impact of tissue hyperemia lasted longer in the contrast therapy group.

### Perfusion unit (PU)

Our results confirm the influence of warm and cold stimuli on changes in tissue perfusion. Still, in the case of contrast therapy, their effect was more significant and lasted longer. It should be noted that when cold and warm stimuli are used, microcirculation vessels have variable reaction mechanisms. In the case of heat, these vessels dilate, and the flow slows down, which is associated with an increase in filtration pressure and the migration of oxygen-rich substances to the muscle cells. In the case of cold stimuli, in the first stage (we observed it in the PostTh5min reaction), vasoconstriction occurs, and perfusion decreases, which after some time becomes hyperemic (PostTh24). From this perspective, the use of cold may seem more beneficial in the case of injured or inflammatory muscles, but in the case of healthy and only overloaded muscles, heat and contrast (in the context of our results) seem to have a more beneficial effect. Increased perfusion also improves muscle tone. However, the conclusions from our results in this respect need to be clarified. Microcirculation plays a crucial role in muscle regeneration by facilitating the delivery of oxygen, nutrients, and growth factors to regenerate muscle fibers and removing metabolic waste products^58^. Muscle regeneration is a complex process involving the activation, proliferation, and differentiation of satellite cells, which are muscle stem cells located between the basal lamina and sarcolemma of muscle fibers^59^. During physical exercise, an inflammatory reaction occurs, and blood vessels may leak, leading to the recruitment of immune cells and activation of resident satellite cells. This pumping action may enhance circulation and promote the removal of metabolic waste products more efficiently than hot or cold therapy alone^48^. Contrast therapy can modulate microcirculation by altering blood flow dynamics through vasodilation and vasoconstriction^40^. This modulation of blood flow may contribute to the therapeutic benefits of contrast therapy in promoting tissue healing, reducing inflammation, and improving recovery from musculoskeletal injuries^60^. However, individual responses to contrast therapy may vary, and it is essential to consider factors such as the specific condition being treated, the duration and intensity of treatment, and the patient's tolerance to temperature changes.

Various interventions, such as heat or cold stress, can elicit diverse hemodynamic responses and improve these physiological capacities^17,60^. Kim et al., confirmed the increased blood flow measured with LDF by postulating that hyperaemic responses facilitate the recovery process by promoting nutrient delivery^61^. It should be noted that some authors have suggested that skin perfusion as measured by LDF may have a counterpart in the responses of muscle hyperemia^48^. Akasaki et al., demonstrated through an animal experiment that repeated thermal therapy led to increased eNOS protein expression, enhanced blood flow, and higher capillary density in the ischaemic hind limb of mice^62^. The endothelium plays a pivotal role in the mechanism of local microvascular autoregulation, serving as a source for various mediators. Among these, NO and prostacyclin act as potent vasodilators, while EDCF2 and endothelin (EDCF1) function as robust vasoconstrictors^63^.

### Muscle tone, elasticity, and stiffness of muscle

In the scientific literature, optimal muscle biomechanical properties, such as muscle tension and elasticity, are not only part of an athlete's functioning^64^ but are crucial. They play a key role in increasing performance^65^, reducing the metabolic costs of movement^66^, storing and releasing elastic energy in cyclic movements^64^, and preventing injuries^67^.

The results of our study suggest that muscle elasticity most effectively returned to resting values immediately after compression contrast therapy. Still, after 24 h, the results were similar to heat therapy. It is widely recognized that hot and cold thermal stimuli lead to changes in muscle hyperaemia, effectively reducing muscle tension and restoring muscle elasticity^68^. Previous research has suggested that a musculotendinous system with more excellent elasticity (less stiffness) can lengthen to absorb external forces and moderately affect energy production during movement^69^. In situ and in vivo studies indicate that tendon fibres surrounding muscles can delay this lengthening during energy-dissipating events, temporarily absorbing impact energy and then releasing it to work on the muscle bundles. This complex mechanism relies on the appropriate control of neurons and blood distribution through microcirculation and is essential in the recovery process^16^. It is widely recognized that hot and cold thermal stimuli lead to changes in muscle congestion, effectively reducing muscle tension while improving muscle stiffness and elasticity^68^.

Although the exact mechanisms behind the effects of warm, cold, and compression stimuli on muscle tone are not fully understood, it is assumed that non-myogenic regulation of muscle tone, associated with increased perfusion, also contributes to the observed improvement in muscle tone^70^. Our research also confirmed such effects. While cold increased muscle tension immediately after the intervention, heat, and contrast therapy reduced it, especially after the increase in tension after physical exercise. The putative physiological mechanism suggests that elevated cytosolic Ca2 + concentrations resulting from impaired perfusion and subsequent tissue hypoxia may induce muscle contraction by activating myosin light chain phosphorylation and subsequent actomyosin cross-bridging, increasing tension^71^. Using contrast therapy or other hot and cold therapies after physical exercise can eliminate the adverse effects of mechanical damage to cell membranes, activate anti-inflammatory processes^40^, and reduce increased muscle soreness^41^. The proper functioning of the neuromuscular system results in an optimal state of muscle tension, which affects the strength and condition of the muscles^16,72^.

### Maximum forearm muscle force (Fmax)

Excessive load on skeletal muscles may cause reduced activity of the neuromuscular system and, as a result, a decrease in muscle strength and power^73^. Combat sports require high power and strength demands, and well-trained athletes likely have neuromuscular adaptations that increase the efficiency of energy delivery from anaerobic pathways^1^. In the scientific literature, the results assessing the impact of heat and cold methods and combination therapies on the recovery rate of strength and power after exercise could be more transparent^39^. Our results are also inconclusive. While the results were statistically significant in the period immediately after exercise and confirmed the effectiveness of all forms of therapy, we did not observe this effectiveness after 24 h. The reason for this situation was that only one recovery session took place. There is evidence to suggest that accelerated enzymatic reactions^74^, faster transport of metabolic products^48^, and elimination of oxidative stress products through microcirculation^75^ are responsible for the rate of recovery of strength and power after joint fatigue. Some authors suggest that contrast therapy may reduce the adverse effects of exercise-related muscle damage (EAMD), inflammation, and delayed onset muscle soreness (DOMS)^76^, increasing the rate of recovery of muscle strength^77^ improving joint mobility after intense exercise^39^.

### Research limitations and practical application

The study's main limitation was the follow-up time and the need for a more detailed analysis of the biochemical parameters of muscle fatigue. The authors are aware of these limitations. A limitation of the study is the short-term evaluation with the lack of repeated sessions of muscle fatigue. Therefore, the study results should be taken care of with caution and indicate directions for further research on contrast therapy. Although the measurement tools used objectively assess the impact of contrast therapy, they have their limitations. Most of them do not describe reference values and are very sensitive, which requires skill in using them. In this case, we use the assessment of changes in response to the applied stimulus. It should also be taken into account that contrast therapy is such a popular regeneration method that expectations regarding the effect may influence the level of measured variables. Forty martial arts athletes took part in the study. In subsequent studies, this group should be enlarged, and the observation period of changes should be extended. Additionally, it may be interesting to observe changes compared to other methods of post-exercise muscle recovery and the effect of contrast therapy on recovery between exercise sessions. Future projects should also focus on assessing and comparing the effects of therapy on people with different levels of physical preparation and practicing different sports disciplines. The most important practical implication is that a single 20-min session with warm and cold stimuli and alternating contrast therapy are sufficient to change muscle biomechanical parameters immediately, and the observed changes are most beneficial immediately after fatigue.

## Conclusions

These studies have shown that a single compression therapy session using warm and cold stimuli and alternating contrast therapy can induce immediate changes in muscle tension, elasticity, and tissue perfusion, affecting muscle strength recovery rate. Observations immediately after therapy are particularly interesting. It should be emphasized that the results are unclear, and no conclusions can be drawn based on which form of this therapy is more effective. This effectiveness appears to decrease after 24 h, which may be due to using only one recovery session after muscle fatigue.

## Data Availability

Data used in this article can be obtained from the corresponding author at the reasonable request of a scientist.
